# Supplementary figures and images for: Structure defining of ultrapotent neutralizing nanobodies against MERS-CoV with novel epitopes on receptor binding domain
Source: PLoS Pathog. 2024 Aug 14;20(8):e1012438. doi: 10.1371/journal.ppat.1012438 (PMC11324105; doi:10.1371/journal.ppat.1012438)

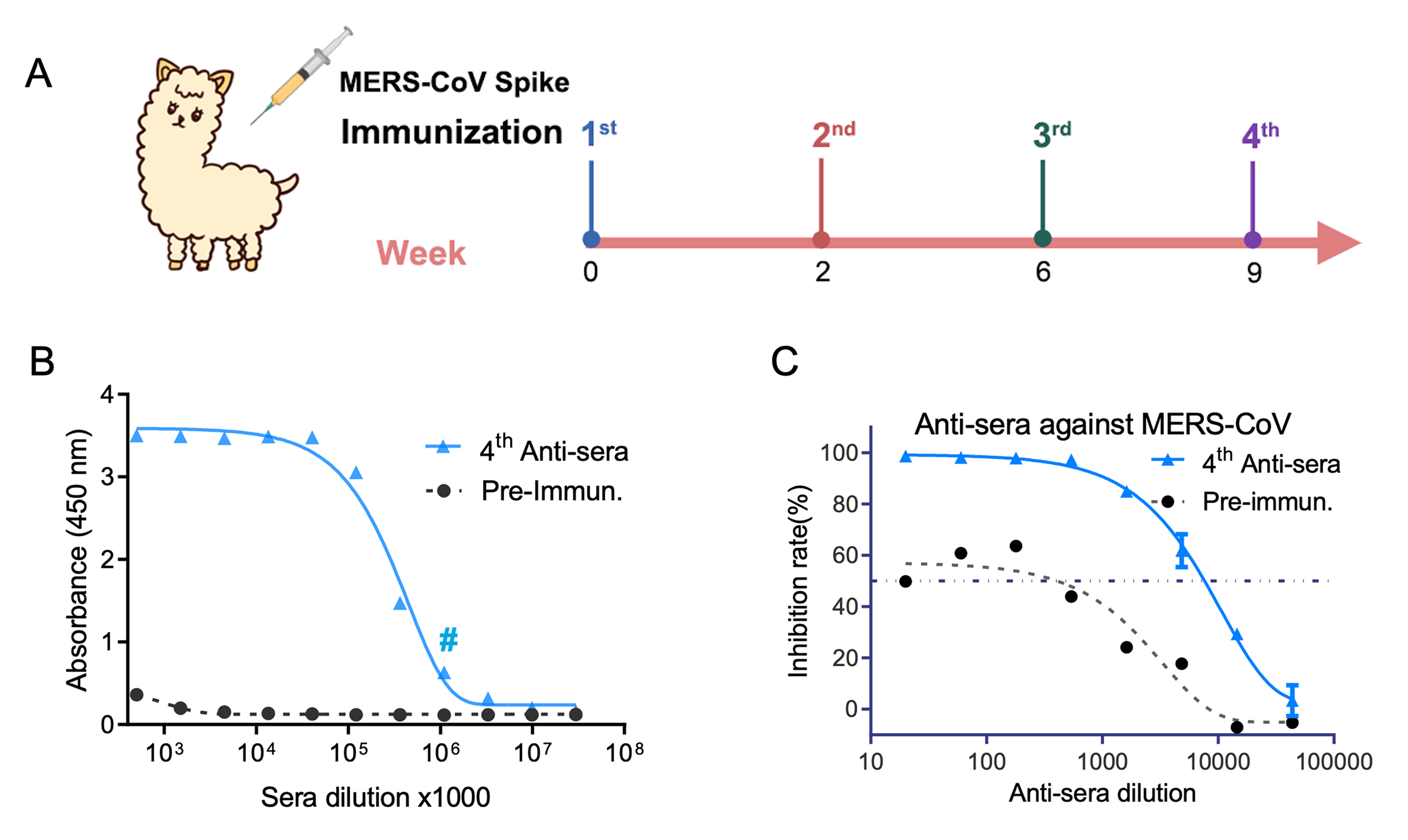

Supplement: S1 Fig — (A). The experimental schedule of immunization. (B). The titer of anti-sera was evaluated by ELSIA after the 4th immunization in alpaca receiving MERS-CoV S protein. Y-axis represented the absorbance at 450 nm, X-axis was the anti-sera dilution fold. 4th anti-sera represented alpaca anti-sera after the 4th immunization of MERS-S protein (cyan line). Pre-Immun. was the sera from the alpaca before the immunization of MERS-CoV S protein. The pound sign indicated the anti-sera titer. (C). Anti-sera inhibited MERS-CoV from infecting Huh-7 cells. The line color was indicated as (B). (TIF) [file ppat.1012438.s001.tif]

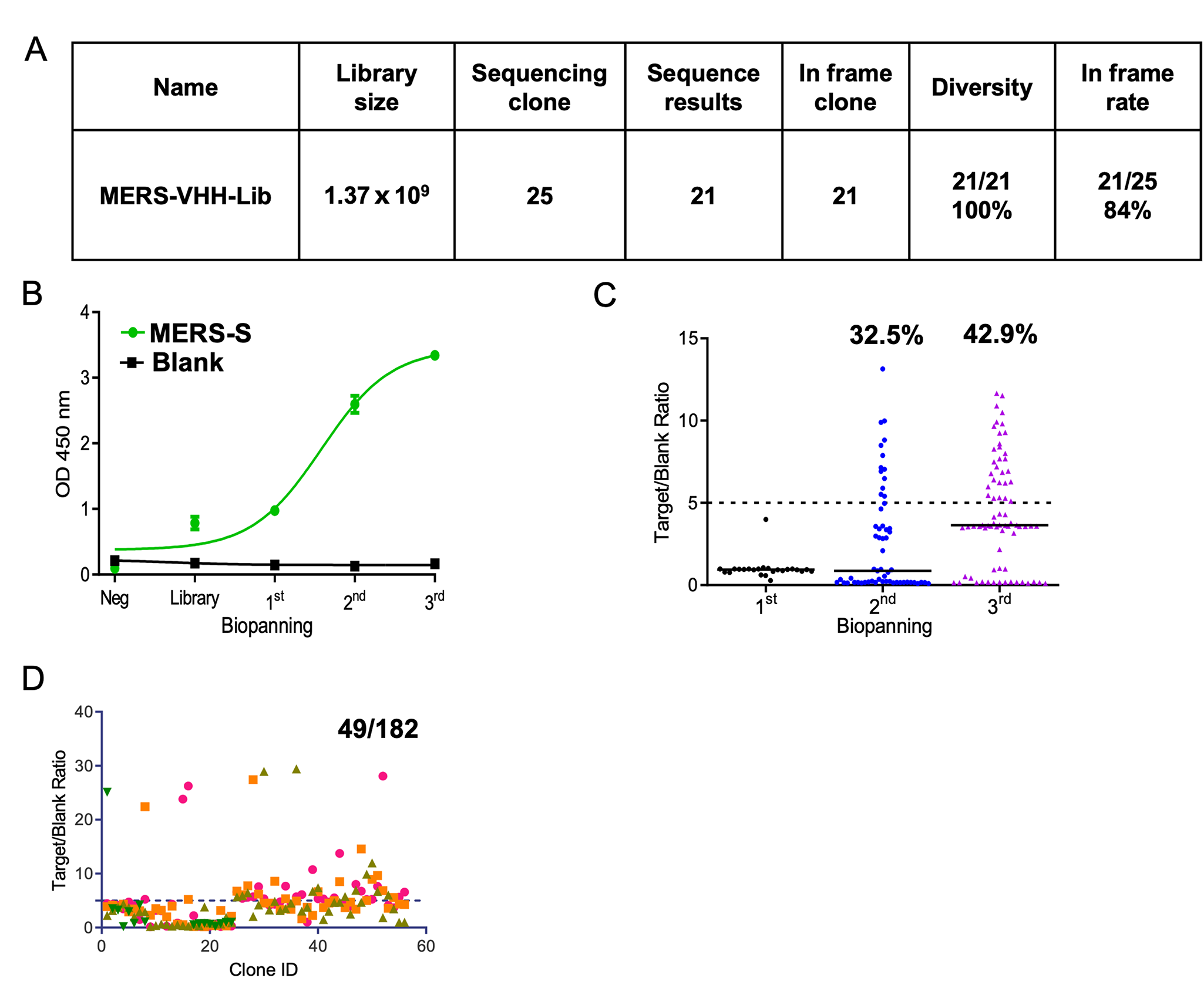

Supplement: S2 Fig — (A). The summary of MERS–VHH-library. (B). The binding of phage library with MERS-CoV S protein identified by phage ELISA, Library is the phage library of MERS-VHH. 1st, 2nd, and 3rd are the phage library after panning on one round, two rounds, and three rounds of MERS-CoV S protein enrichment, respectively. (C). Positive clones of phage from MERS-VHH library after the 1st, 2nd, and 3rd enrichment of MERS-S protein. One dot represented a single clone. (D). The summary of bacterial supernatant binding with MERS- CoV S protein tested by phage ELISA. The Y-axis was the ratio of the readout of MERS-CoV S binding/the readout of blank binding. One dot represented bacterial supernatant from single clone. The ratio above 5 was taken as positive binders. Among 182 clones, there were 49 positive binders. (TIF) [file ppat.1012438.s002.tif]

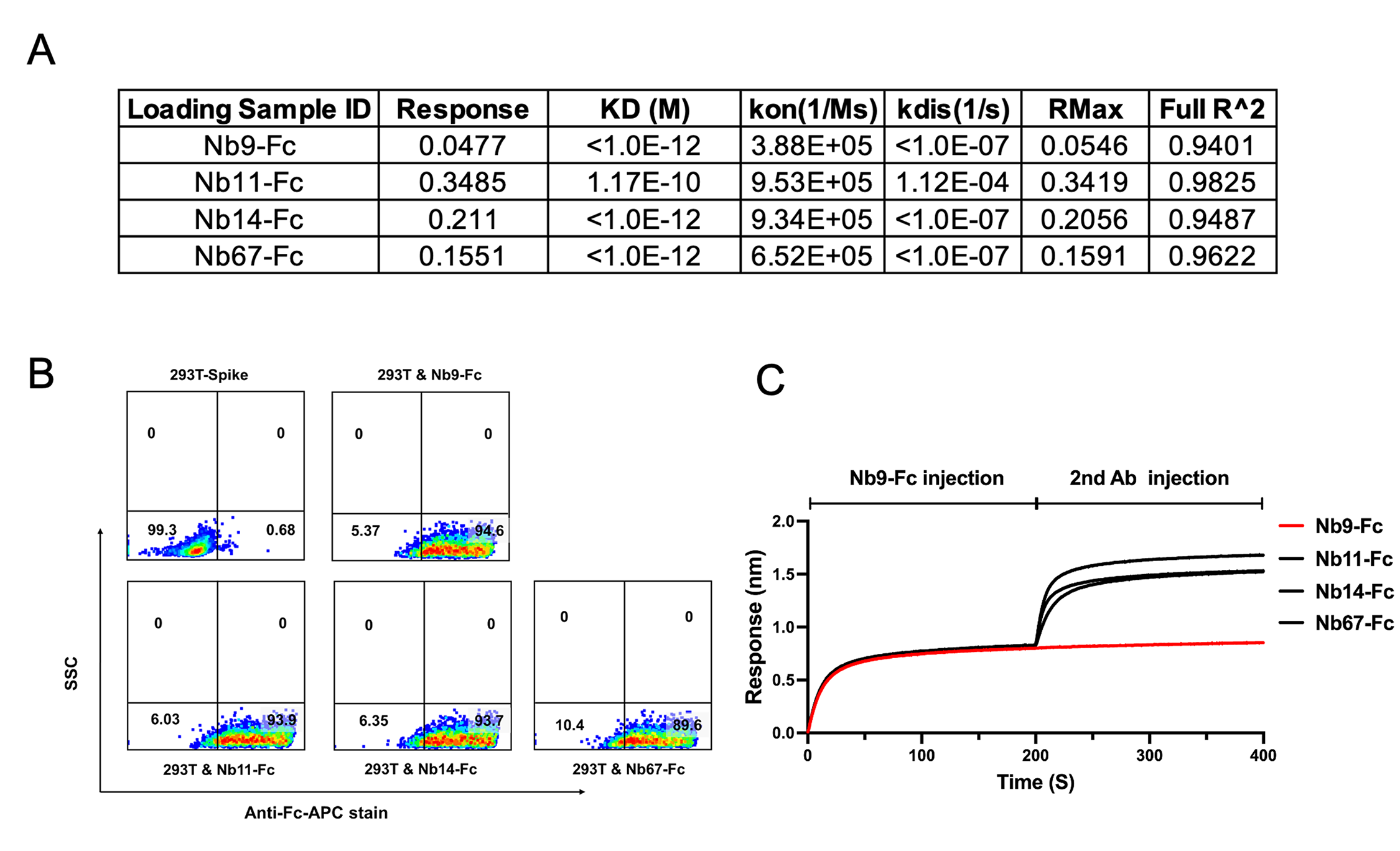

Supplement: S3 Fig — (A). The affinity summary of Nbs binding with MERS-S1 protein tested by BLI. Kon, association rate constant, kd, dissociate constant; KD, binding affinity; RMax, the max response unit. (B). Nb-Fcs binding with MERS-CoV S which overexpressed in HEK-293T cells and analyzed by FACS, associated with Fig 2A. (C). Epitope analysis of Nbs by BLI. MERS-S1 protein was coated on the sensor, and Nb9 as the first antibody was added to bind for 200 s, followed by Nb11, Nb14, and Nb67 as the second antibody for another 200 s. (TIF) [file ppat.1012438.s003.tif]

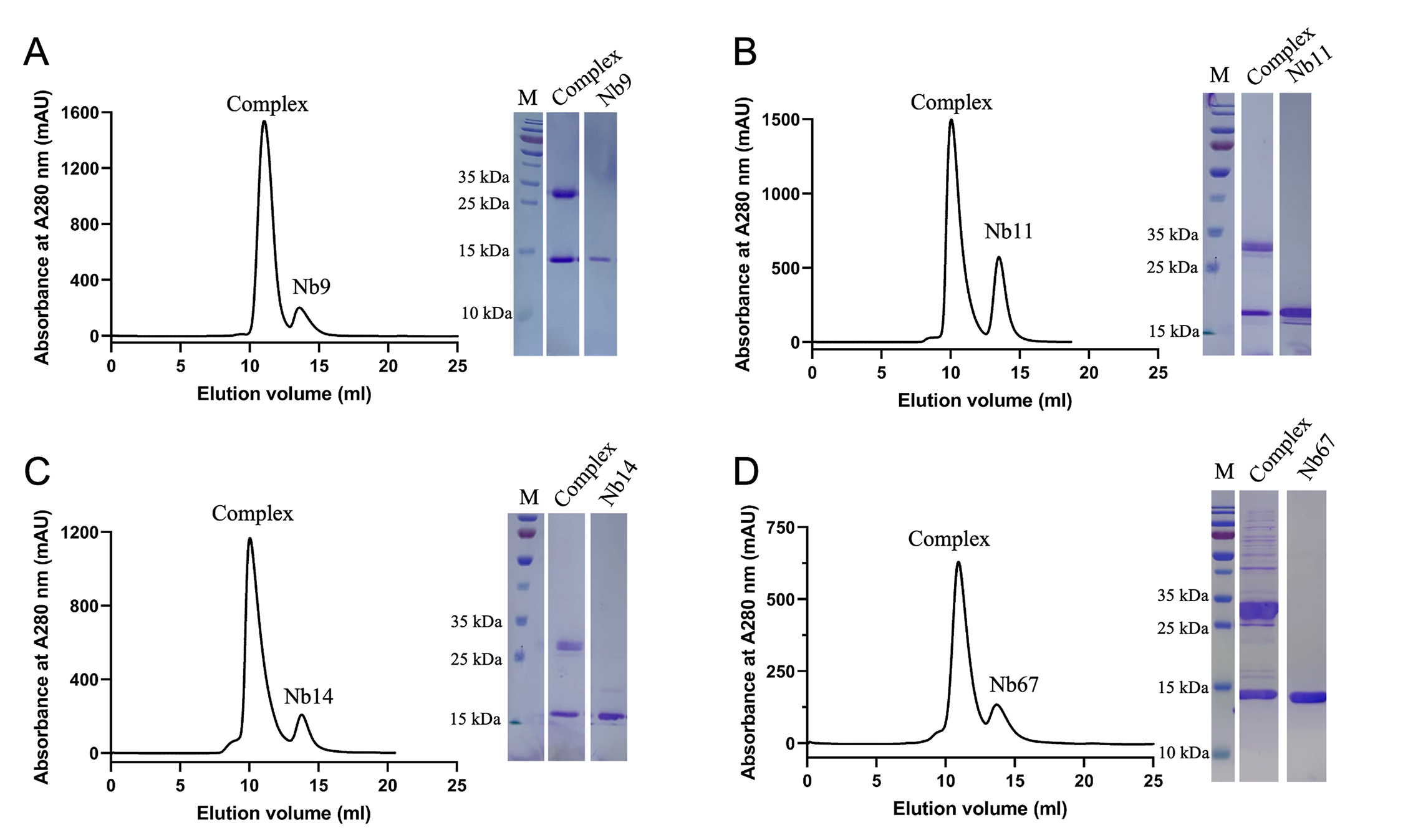

Supplement: S4 Fig — The gel chromatography and SDS-PAGE analyses of RBD and Nb9 (A), Nb11 (B), Nb14 (C) and Nb67 (D). (TIF) [file ppat.1012438.s004.tif]

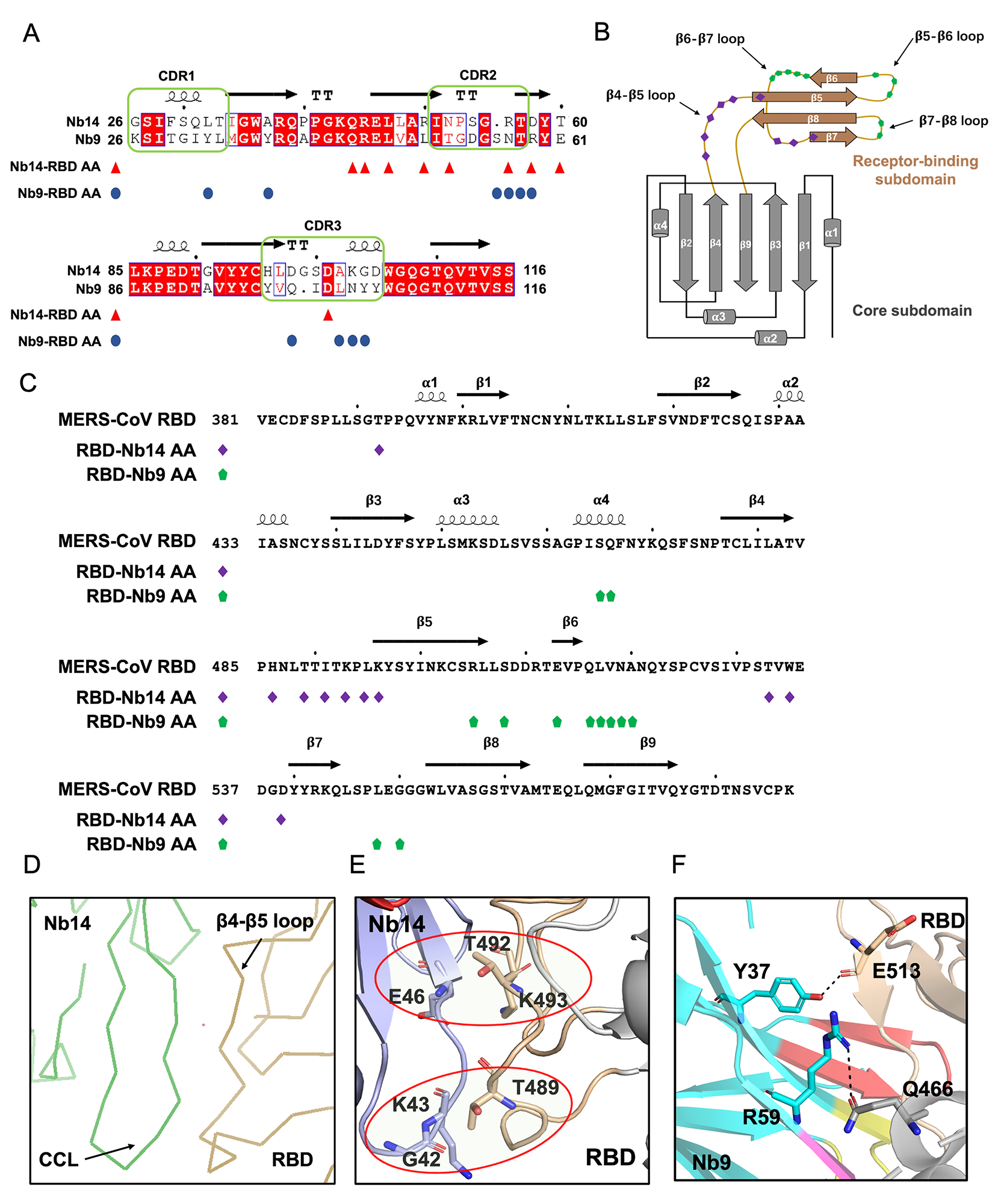

Supplement: S5 Fig — (A). The sequence alignment of Nb9 and Nb14. The red triangles represented the residues of Nb14 interacted with RBD, and the blue dots represented the residues of Nb9 interacted with RBD. (B) and (C). The residues of RBD interacted with Nb9 (blue dots) and Nb14 (red triangles). (D). The CCL loop of Nb14 interacted with the β4-β5 loop of RBD. (E). the hydrophobic network of Nb14 CCL loop and RBD β4-β5 loop. (F). The residues R59 and Y37 of Nb9 FRs formed hydrogen bonds with Q466 and E513 of RBD. (TIF) [file ppat.1012438.s005.tif]

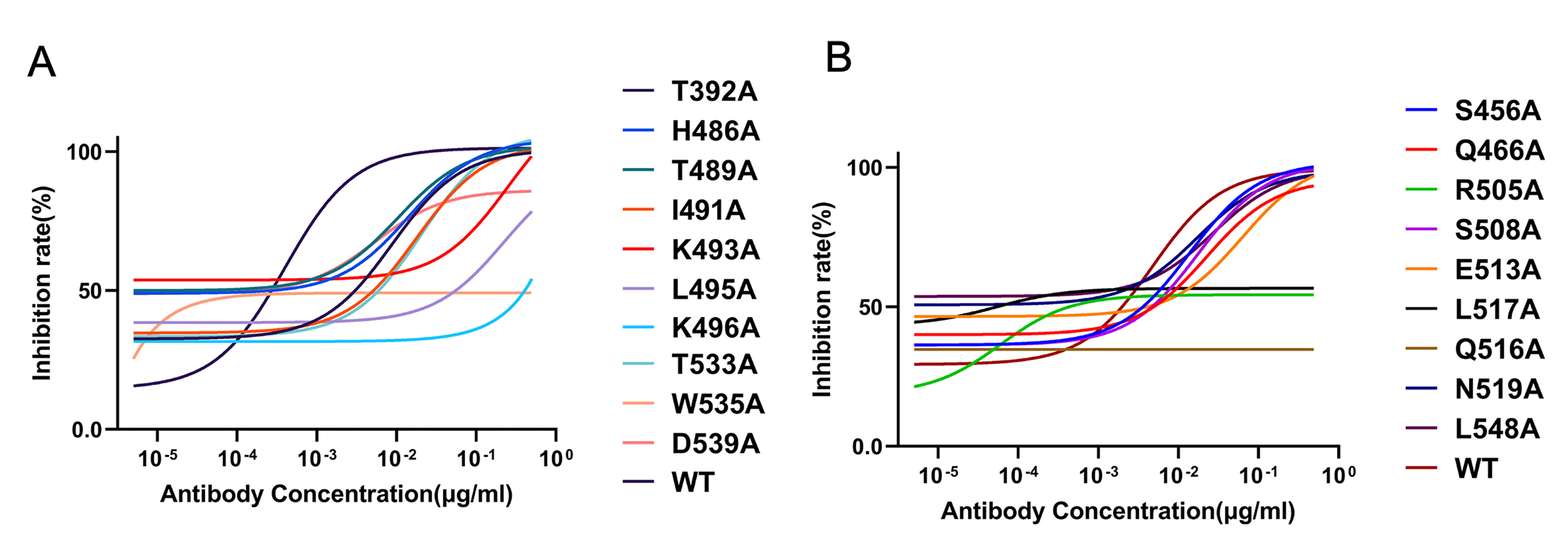

Supplement: S6 Fig — The IC50 of Nb14 (A) and Nb9 (B) against different pseudotyped MERS-CoV with natural mutations. (TIF) [file ppat.1012438.s006.tif]

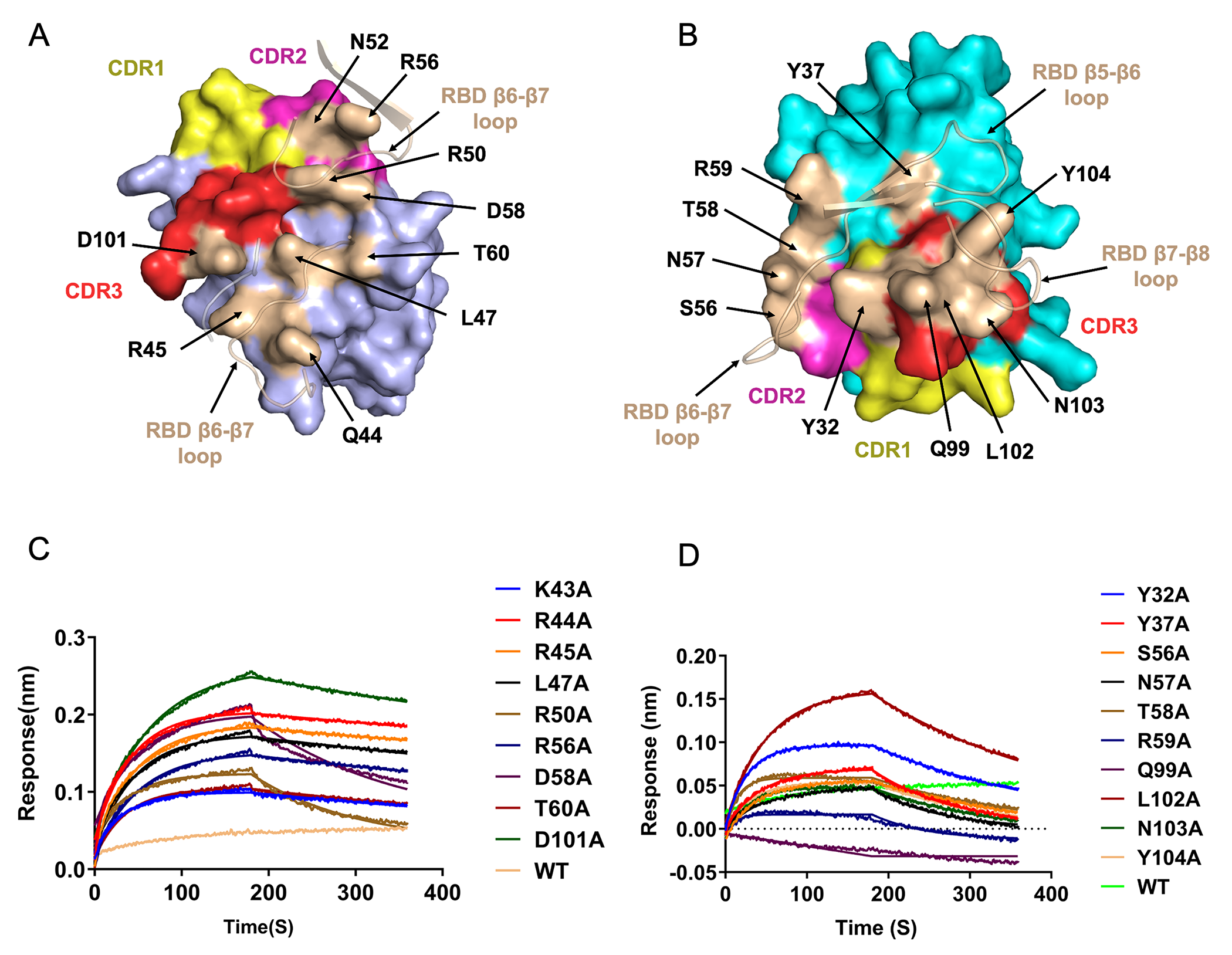

Supplement: S7 Fig — (A). The key residues of Nb14 paratope. (B). The key residues of Nb9 paratope. All the CDRs are shown as yellow (CDR1), violet (CDR2), and red (CDR3). (C). The binding affinities of Nb14 mutations for RBD bound. (D). The binding affinities of Nb14 mutations for RBD bound. Statistics are summarized in S5 Table. (TIF) [file ppat.1012438.s007.tif]

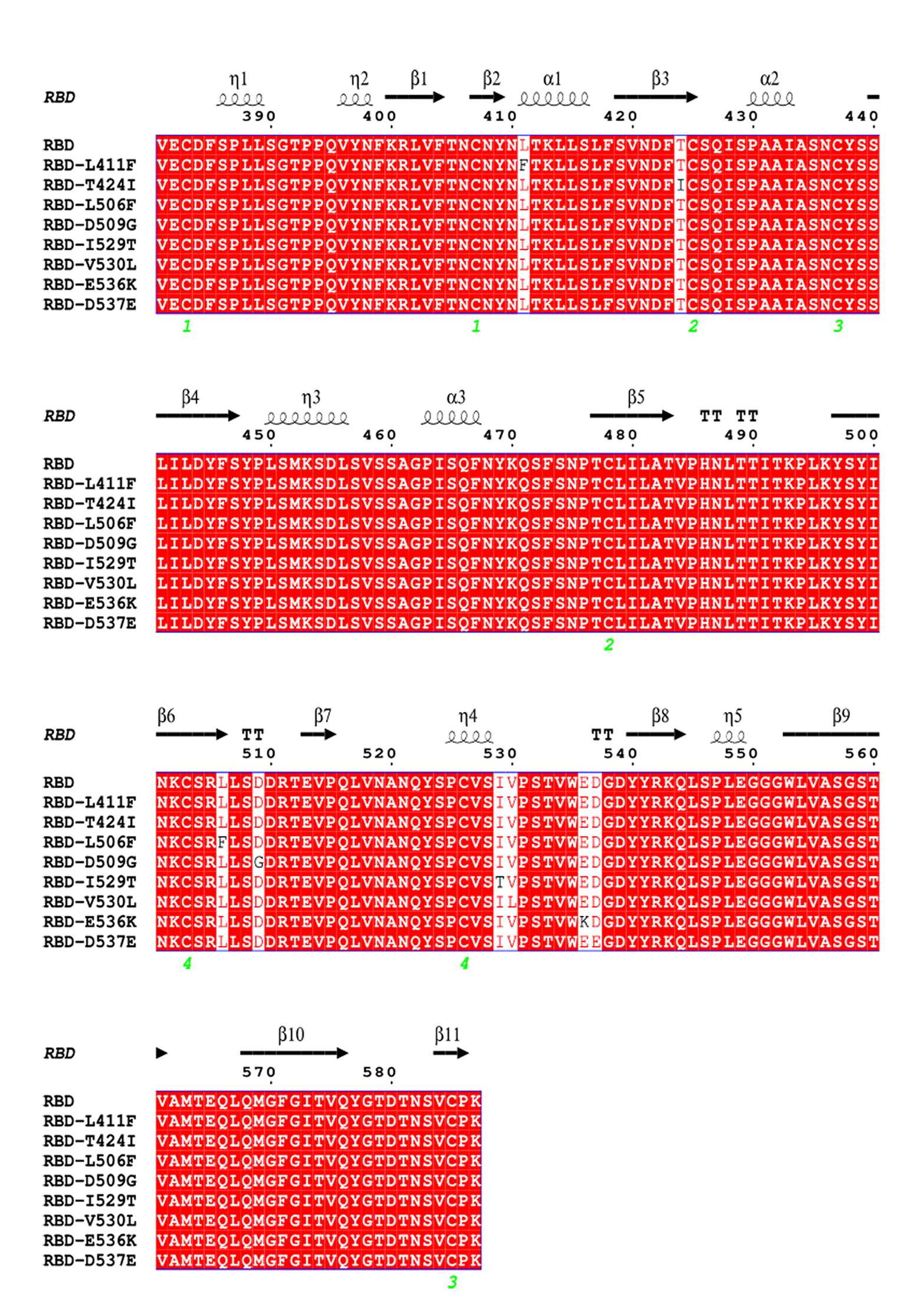

Supplement: S8 Fig — (TIF) [file ppat.1012438.s008.tif]

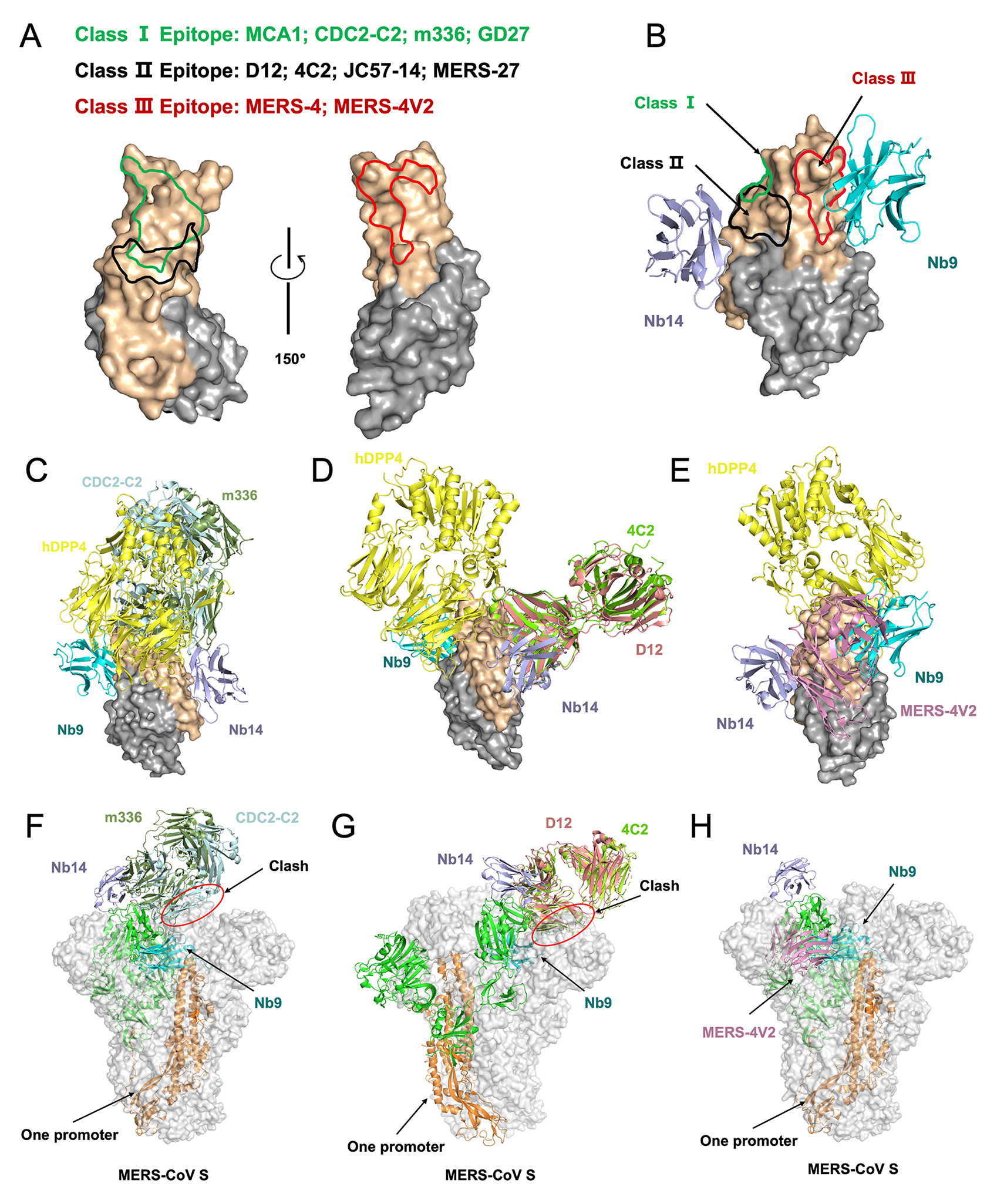

Supplement: S9 Fig — (A). The epitopes of three classes are circled by green (Class I), black (Class II), and red (Class III) rings. (B). The epitope of Nb14 is out of the three Classes. The epitope of Nb9 is partially overlapped with the Class III epitope. (C-E). Structural superimpose between Class I, Class II, and Class III antibodies with hDPP4-RBD. (F-H). Structural superimposed between Class I, Class II, and Class III antibodies with MERS-CoV S which with all RBD in the “down” positions. CDC2-C2 (PDB: 6C6Z); m336 (PDB: 4XAK); 4C2 (PDB: 5DO2); D12 (4ZPT); MERS-4V2 (PDB: 5YY5); hDPP4: (4L72); MERS-CoV S (PDB: 5w9j). (TIF) [file ppat.1012438.s009.tif]

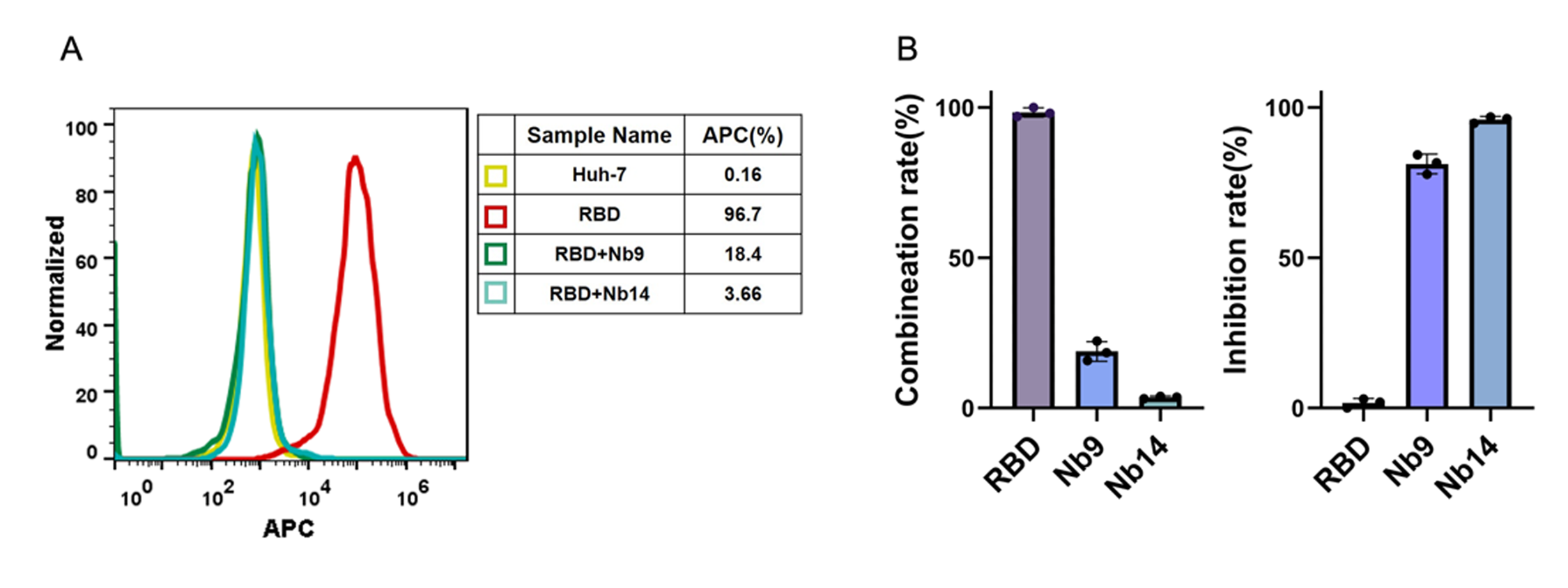

Supplement: S10 Fig — (A). Nb14 and Nb9 inhibit the binding of RBD and Huh-7 cells, and the FACS peak shifts forward. RBD: Red; RBD+Nb14: green; and RBD+Nb9: cyan. (B). The histogram results of Nb14 and Nb9 inhibition. Data are represented as mean ± SD from three independent experiments. (TIF) [file ppat.1012438.s010.tif]

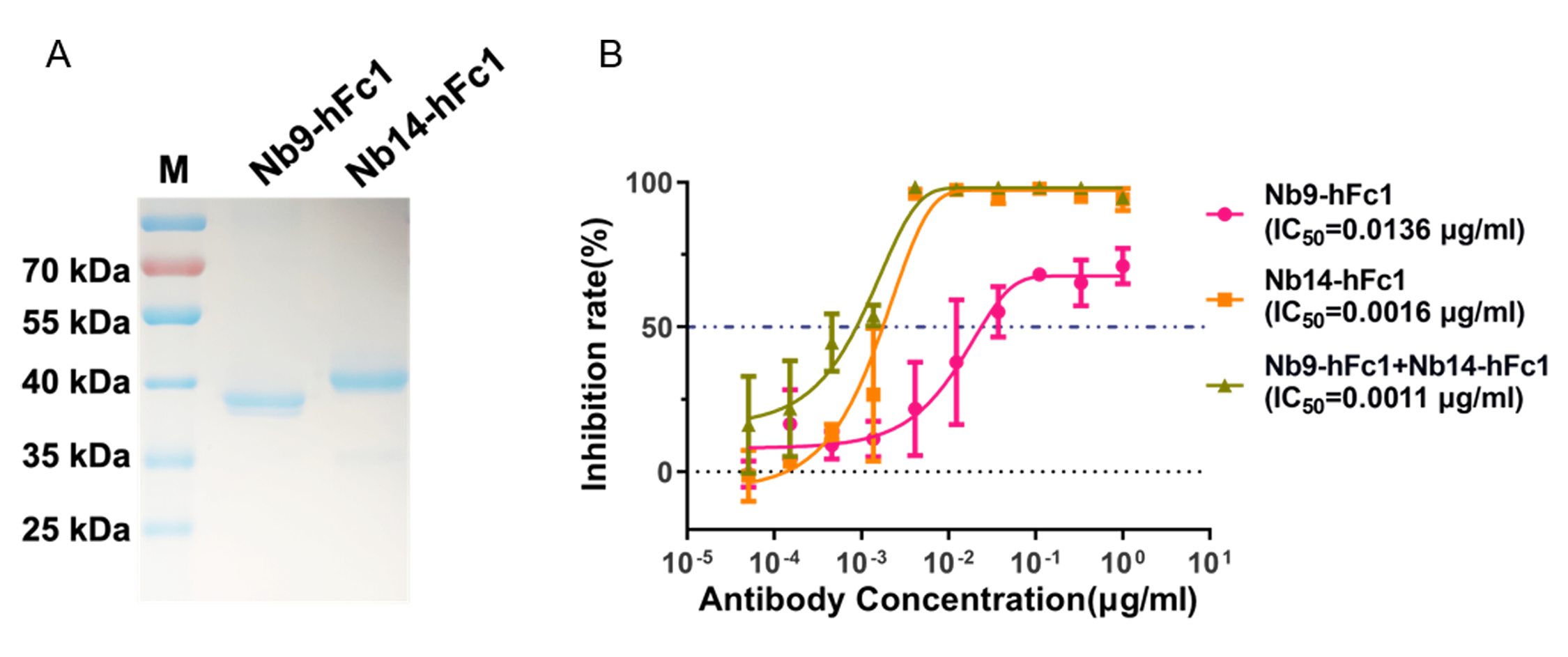

Supplement: S11 Fig — (A). The SDS-PAGE analyses of Nb9-hFc1 and Nb14-hFc1. (B). Neutralizing effects of Nb14-hFc1 combined with Nb9-hFc1 against pseudotyped MERS-CoV. (TIF) [file ppat.1012438.s011.tif]
