# Supplementary material for: Structure defining of ultrapotent neutralizing nanobodies against MERS-CoV with novel epitopes on receptor binding domain
Source: PLoS Pathog. 2024 Aug 14;20(8):e1012438. doi: 10.1371/journal.ppat.1012438 (PMC11324105; doi:10.1371/journal.ppat.1012438)
Supplement: Attachment — Submitted filename: review.docx [file ppat.1012438.s018.docx]

Ma, et al, studied four potent nanobodies against MERS-CoV isolated from alpaca. Therein, Nb14 and Nb9 targets the different cryptic face of RBD, and that Nb14 framework regions are mainly involved in interactions that target a novel epitope, which is entirely distinct from all three class antibodies. The discovery of these Nbs holds great promise in clinical therapeutics, offering innovative solutions in response to the persistent and evolving challenges posed by coronaviruses. It is a topic of interest to the researchers in the related areas but the paper needs very some improvement before acceptance for publication.

My detailed comments are as follows:

1. P9: “The library had a size of 1.37 × 10^^9^, exhibiting a remarkable sequence diversity of 100% with an impressive in-frame rate of 84%, which was rigorously validated through PCR and sequencing.”

10^^9^ should be 10^9^ for consistency.

2. P10 and P11: “The neutralizing activity of Nbs was dosedependent in Huh-7 cells, and with stronger potency of Nb14 (IC_50_, 0.0014 μg/ml), Nb11 (IC_50_, 0.0022 μg/ml), and Nb67 (IC_50_, 0.0025 μg/ml), respectively (Fig. 2E and 2H). In contrast, Nb9 displayed a slightly weaker neutralizing activity with an IC_50_ of 0.016 µg/ml (Fig. 2E and 2H).”

According to Fig. 2H, the IC_50_ of Nb11 should be 0.0023 μg/ml and the IC_50_ of Nb9 should be 0.0165 μg/ml.

3. P19: “The FACS showed that Nb9 and Nb9+Nb14 inhibition rates, which blocked the staining of Huh-7 cells by the RBD, were 80% and 97%, respectively (Fig. 8B).”

According to Fig. 8B, the inhibition rates of Nb9+Nb14 is about 93%?

How about the inhibition rates of Nb14 alone? According to Fig. 6B, the inhibition rates of Nb14 is about 93% and is close to the inhibition rates of Nb9+Nb14. In this way, dose these results fully illustrate that Nb14 could cooperate with Nb9 due to differences in epitopes?
