# Supplementary material for: Structure defining of ultrapotent neutralizing nanobodies against MERS-CoV with novel epitopes on receptor binding domain
Source: PLoS Pathog. 2024 Aug 14;20(8):e1012438. doi: 10.1371/journal.ppat.1012438 (PMC11324105; doi:10.1371/journal.ppat.1012438)
Supplement: Attachment — Submitted filename: Response to Reviewers.docx [file ppat.1012438.s019.docx]

Dear Editor，

Thank you for your decision letter concerning our manuscript (PPATHOGENS-D-24-01034) entitled "Structure defining of ultrapotent neutralizing nanobodies against MERS-CoV with novel epitopes on receptor binding domain", and your time regarding for our revision. I also appreciate all the critical comments from you and reviewers. We have carefully considered the comments and revised the manuscript accordingly. With these improvements, we hope that the current version can meet the Journal’s standards for publication. The following is a point-by-point response to all those comments and a list of changes we have made to the manuscript.

Sincerely

Yaxin Wang

**Point-by-point responses to the comments of the Editor and reviewers, and a list of changes are:**

Reviewer: 1

Comments to the Author

Ma, et al, studied four potent nanobodies against MERS-CoV isolated from alpaca. Therein, Nb14 and Nb9 targets the different cryptic face of RBD, and that Nb14 framework regions are mainly involved in interactions that target a novel epitope, which is entirely distinct from all three class antibodies. The discovery of these Nbs holds great promise in clinical therapeutics, offering innovative solutions in response to the persistent and evolving challenges posed by coronaviruses. It is a topic of interest to the researchers in the related areas but the paper needs very some improvement before acceptance for publication.The study provides valuable insights into DENV infection, host response, as well as the potential development of innovative antiviral strategies. I have a few minor suggestions listed below.

Response: We would like to thank you for your professional review work, constructive comments, and valuable suggestions on our manuscript. Following your comments, we revise our manuscript and provide point-by-point answers to the your questions as follow.

Minor Issues:

1. P9: “The library had a size of 1.37 × 10^9, exhibiting a remarkable sequence diversity of 100% with an impressive in-frame rate of 84%, which was rigorously validated through PCR and sequencing.” 10^9 should be 109 for consistency.

Response: We have corrected it accordingly.

2. P10 and P11: “The neutralizing activity of Nbs was dosedependent in Huh-7 cells, and with stronger potency of Nb14 (IC_50_, 0.0014 μg/ml), Nb11 (IC_50_, 0.0022 μg/ml), and Nb67 (IC_50_, 0.0025 μg/ml), respectively (Fig. 2E and 2H). In contrast, Nb9 displayed a slightly weaker neutralizing activity with an IC_50_ of 0.016 µg/ml (Fig. 2E and 2H).” According to Fig. 2H, the IC_50_ of Nb11 should be 0.0023 μg/ml and the IC_50_ of Nb9 should be 0.0165 μg/ml.

Response: Thank you for your careful review, we have corrected this error and highlighted it in yellow.

3. P19: “The FACS showed that Nb9 and Nb9+Nb14 inhibition rates, which blocked the staining of Huh-7 cells by the RBD, were 80% and 97%, respectively (Fig. 8B).”

According to Fig. 8B, the inhibition rates of Nb9+Nb14 is about 93%?

How about the inhibition rates of Nb14 alone? According to Fig. 6B, the inhibition rates of Nb14 is about 93% and is close to the inhibition rates of Nb9+Nb14. In this way, dose these results fully illustrate that Nb14 could cooperate with Nb9 due to differences in epitopes?

Response: Thank you for this valuable comment. We have corrected the mistake (97% to 93%). The inhibition rate of Nb14 alone is 96%. When Nb14+Nb9, the inhibition rate is 98%. Since Nb14 almost completely neutralized the RBD when used alone, synergistic neutralization effect was not significantly improved after adding Nb9 (96% shift to 98%). This data was not shown in the manuscript (see the following Fig. R1). On the contrary, the inhibition effect of Nb9 is slightly less, and the synergistic effect is very apparent after adding Nb14 (80% shift to 93%). These results could illustrate that Nb14 cooperate with Nb9 due to the difference in epitopes.

Figure R1. The synergistic neutralization effect of Nb14 alone, and firstly incubated Nb14 with RBD and then added Nb9.

Reviewer: 2

Comments to the Author

In this manuscript, Ma et al, utilized the recombinant MERS-CoV S protein to immunize the alpaca and successfully isolated four nanobodies with ultrapotent neutralizing activity. The authors further defined the crystal structures of Nb14-/Nb9-RBD and elucidated the molecular mechanism of how Nb14 and Nb9 work. Notably, among these four nanobodies, Nb14 exhibited the most prominent activity and specifically targeted a novel epitope. This study provides a highly valuable research basis for the combination of two nanobodies against MERS-CoV, leveraging the distinct recognition epitopes and mechanisms of action exhibited by Nb14 and Nb9, and provides insights for the development of broad-spectrum nanobodies against MERS-CoV. Overall, this work is very interesting and important. This reviewer believe this manuscript merits publication, if the authors can address the following concerns.

Response: We thank the reviewer for the positive and constructive comments regarding our paper.

Major Issues:

1. The authors evaluated the synergistic effect of the combination of Nb14 and Nb9 based on the different epitopes. Nanobody traditionally requires fusing human Fc to extend the half-life in the human body. Therefore, will Nb14 and Nb9 retain the same synergistic effect when fused with Fc? The authors should test, or at least discuss this issue in their revision.

Response: Thanks to your suggestions. We have tested the synergistic effect of the combination of Nb14-Fc and Nb9-Fc. The results indicated that human Fc did not effect the combination of these two nanobodies (see S11 Fig)

2. In Figure 6B, the FACS results should be consistent with that of Figure 8B, and the data of different experimental groups should be counted as a histogram.

Response: We added the histogram for Figure 6B (see S10 Fig).

Minor Issues:

1. In Figure 5, the authors evaluated the neutralizing activity of Nb14 and Nb9 against MERS-CoV strains with several natural mutations. The authors should add a sequence alignment diagram to show the location of each mutation on RBD of MERS-CoV strains.

Response: The reviewer’s points are well taken. We have added the sequence alignment of different MERS-CoV RBDs in S8 Fig.

2. “We similarly compared the binding of Class I, Class II, and Class III antibodies to the closed state of MERS-CoV S by structure superimpose.” In this part, the “MERS-CoV S” means the S trimer?

Response: The “MERS-CoV S” means the S trimers. We have corrected this sentence.

3. “Although we could not character the atom level” should be “atomic level”.

Response: We have corrected it.

4. “Statistics on diffraction data collection” should be “Statistics of”.

Response: We have corrected this sentence.

5. “CDR2 and CDR3 of Nb14 binding with RBD” should be “are binding”.

Response: We have corrected the grammar of this sentence.

6. “Particularly, these atural changes, named L506, D509, and E536”, the “atural” should be “natural”.

Response: We have corrected this error.

7. “Binding the hDPP4 host receptor and activating the S trimer is necessary for virus infection”, the “S trimer” should be “S trimers”.

Response: We have corrected this mistake.

8. “Our findings indicate that while most antibodies exhibited limited effectiveness in binding to the closed-state MERS-CoV S”. In this section, the “MERS-CoV S” expression should be “MERS-CoV S trimers”, consistent with the previous.

Response: We have corrected this sentence.

9. “The MERS-CoV NTD and RBD were express Bac-to-Bac baculovirus system” should be “were expressed”.

Response: We have corrected the grammar of this sentence.

10. “Nb14 were added into one of the mixtures and incubated at 37 ℃ for 30 min” should be “Nb14 was added”.

Response: We have corrected the grammar of this sentence.

11. Figure 2 legends “(E, F). Nb9, Nb11, Nb14, and Nb67could effectively neutralize MERS-CoV pseudovirus in vitro” the “Nb67could” should be “Nb67 could”.

Response: We have added space between words.

Reviewer: 3

Comments to the Author

Ma et al. describe the identification and characterization of four nanobodies against MERS-CoV. Two of these are studied in detail using a range of techniques to demonstrate unique binding and neutralizing capacities. The mechanisms of neutralization identified, either blocking the RBD in the upward postion or RBD-glycan interactions add to the understanding of the interaction of MERS-CoV spike protein with DPP4. Mutants made, both RBD as well as DPP4, add to the further characterization next to the structural studies. Overall these are intersting findings.

Response: We would like to thank the respected reviewer 3 for all those useful comments.

Major Issues:

1. My major concern is the gramma used. The article should be fully checked. In all sections, including the tittle, abstract as well as the results section many corrections should be made.

Response: We have carefully checked the article and corrected the gramma errors (marked in green).

Minor Issues:

1. Intro: MERS-CoV did not cause a pandemic...

Response: We have modified the expression of sentence.

2. SARS-CoV-1 should be replaced by SARS-CoV

Response: We have changed “SARS-CoV-1” to “SARS-CoV”

3. When analyzing different mutations in the RBD in pseudotypes small drops in neutralizing capacity are observed. However, variation in the IC_50_ values against WT virus are not shown.

Response: The variation in the IC_50_ values against WT virus were shown in the table of Fig 5F (fold changes of Nb9 and Nb14 are 1).

4. page nrs or lines are not provided which makes it difficult to provide accurate feedback...

Response: Thanks for your professional suggestions. We have added the number of pages and lines.
